# Global Phylogeny of *Mycobacterium avium* and Identification of Mutation Hotspots During Niche Adaptation

**DOI:** 10.3389/fmicb.2022.892333

**Published:** 2022-05-06

**Authors:** Rachel Mizzi, Karren M. Plain, Richard Whittington, Verlaine J. Timms

**Affiliations:** ^1^Farm Animal Health, School of Veterinary Science, Faculty of Science, The University of Sydney, Camden, NSW, Australia; ^2^Microbiology and Parasitology Research, Elizabeth Macarthur Agricultural Institute, Menangle, NSW, Australia; ^3^Neilan Laboratory of Microbial and Molecular Diversity, College of Engineering, Science and Environment, The University of Newcastle, Newcastle, NSW, Australia

**Keywords:** *Mycobacterium avium*, *hominissuis*, *paratuberculosis*, *silvaticum*, comparative genomics, mutation hotspot

## Abstract

*Mycobacterium avium* is separated into four subspecies: *M. avium* subspecies *avium* (MAA), *M. avium* subspecies *silvaticum* (MAS), *M. avium* subspecies *hominissuis* (MAH), and *M. avium* subspecies *paratuberculosis* (MAP). Understanding the mechanisms of host and tissue adaptation leading to their clinical significance is vital to reduce the economic, welfare, and public health concerns associated with diseases they may cause in humans and animals. Despite substantial phenotypic diversity, the subspecies nomenclature is controversial due to high genetic similarity. Consequently, a set of 1,230 *M. avium* genomes was used to generate a phylogeny, investigate SNP hotspots, and identify subspecies-specific genes. Phylogeny reiterated the findings from previous work and established that *Mycobacterium avium* is a species made up of one highly diverse subspecies, known as MAH, and at least two clonal pathogens, named MAA and MAP. Pan-genomes identified coding sequences unique to each subspecies, and in conjunction with a mapping approach, mutation hotspot regions were revealed compared to the reference genomes for MAA, MAH, and MAP. These subspecies-specific genes may serve as valuable biomarkers, providing a deeper understanding of genetic differences between *M. avium* subspecies and the virulence mechanisms of mycobacteria. Furthermore, SNP analysis demonstrated common regions between subspecies that have undergone extensive mutations during niche adaptation. The findings provide insights into host and tissue specificity of this genetically conserved but phenotypically diverse species, with the potential to provide new diagnostic targets and epidemiological and therapeutic advances.

## Introduction

The *Mycobacterium avium* complex (MAC) is a group of slow-growing (>1 week to form visible colonies during culture) non-tuberculosis mycobacteria (NTM). A recent definition of these species was described by van Ingen et al. (2018). They characterized MAC species by a sequence identity of >99.4% for the full *16S* rRNA gene, >97.3% for *hsp65*, and > 94.4% for *rpoB* region V for reference stains *Mycobacterium intracellulare* ATCC 13950 (Kim et al., 2012) or *Mycobacterium avium* ATCC 25291 (Goethe et al., 2020). According to this definition, the MAC contains 12 species: *Mycobacterium avium* (Thorel et al., 1990), *Mycobacterium intracellulare* (Wayne et al., 1993), *Mycobacterium chimaera* (Tortoli et al., 2004), *Mycobacterium colombiense* (Murcia et al., 2006), *Mycobacterium arosiense* (Bang et al., 2008), *Mycobacterium vulneris* (van Ingen et al., 2009), *Mycobacterium bouchedurhonense*, *Mycobacterium timonense*, *Mycobacterium marseillense* (Ben Salah et al., 2009), *Mycobacterium yongonense* (Kim et al., 2013), *Mycobacterium paraintracellulare* (Lee et al., 2016a), and *Mycobacterium lepraemurium*.

**Figure 1:**
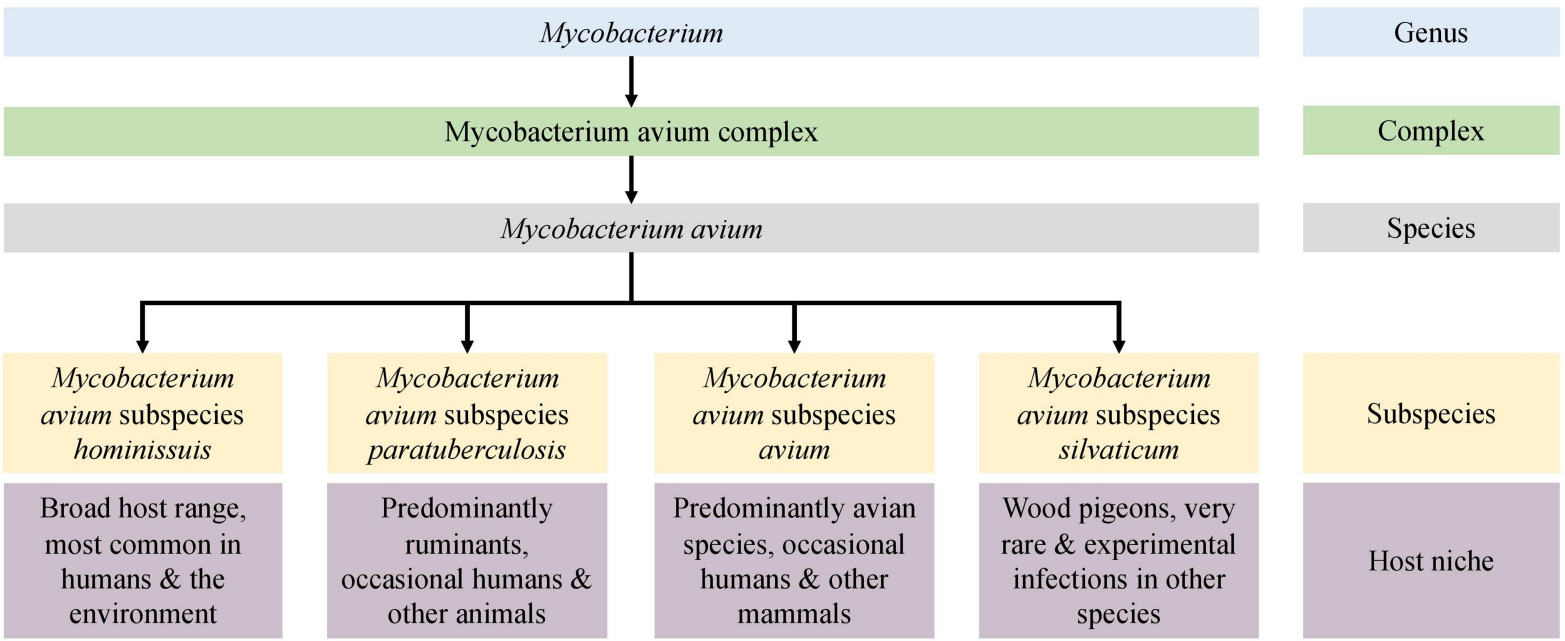
An overview of the *Mycobacterium avium* subspecies.

*Mycobacterium avium* has been separated into four subspecies: *M. avium* subspecies *avium* (MAA)*, M. avium* subspecies *silvaticum* (MAS), *M. avium* subspecies *hominissuis* (MAH), and *M. avium* subspecies *paratuberculosis* (MAP; Figure 1). Understanding the clinical significance and mechanisms of host and tissue adaptation of these subspecies is vital to reduce economic, welfare, and public health concerns associated with the diseases they cause in humans and animals. Despite the obvious phenotypic diversity of these subspecies, their nomenclature is controversial due to their high degree of genetic similarity. Furthermore, little is known about the biological reasons for different *M. avium* subspecies to infect and survive in different host and tissue niches.

Early descriptions and infection trials demonstrated differences in the pathogenicity and host range of ruminant and avian mycobacterial isolates, leading to the hypothesis that there were several *M. avium* subspecies (Collins et al., 1985). In 1990, three *M. avium* subspecies were recognized (Thorel et al., 1990), with the former species *M. paratuberculosis* being included as a subspecies and named *M. avium* subsp. *paratuberculosis* (MAP). Differences between human and porcine isolates and avian strains were identified using molecular methods, and this led to the nomination of MAH for these *M. avium* isolates (Mijs et al., 2002). MAP is arguably the most studied pathogen in the *M. avium* complex and is the causative agent of paratuberculosis or Johne’s disease (JD), a chronic gastroenteritis that predominately affects ruminants. This pathogen has also been implicated in the pathogenesis of Crohn’s disease (Bach, 2015; Waddell et al., 2015; Timms et al., 2016), type 1 diabetes, and multiple sclerosis (Eslami et al., 2019; Ekundayo et al., 2022) in humans. The tissue tropism of MAP in ruminants is the gastrointestinal tract, specifically the ileum, though disseminated infection to other organs and tissues occurs as the disease progresses. This primary site of infection is unique to this subspecies; other subspecies tend to preferentially infect the respiratory tract or are acute disseminated infections with no specific tissue preference.

*Mycobacterium avium* subspecies *avium* and MAS typically cause respiratory disease in avian species, with the latter almost exclusively restricted to wood pigeons (*Columba palumbus*). Tuberculosis-like respiratory disease caused by MAA in avian species is a common disease that can be economically important due to high mortalities and has welfare concerns, but it is less often reported in the literature than JD in ruminants (Moravkova et al., 2013; Salamatian et al., 2020). MAS are the least reported of the subspecies, and little is known about this mycobacterium due to the relatively small number of isolates available for study.

*Mycobacterium avium* infections reported in humans and swine are typically caused by MAH. In humans, cases typically present as pulmonary disease in immunocompetent individuals, peripheral lymphadenopathy in children, or disseminated infection in immunocompromised patients (Slany et al., 2016). Cases in immunocompetent individuals are particularly concerning due to the high prevalence of antimicrobial resistance of among *M. avium* isolates, particularly MAH (Brown-Elliott et al., 2012; Wang et al., 2021). In swine, mesenteric, cranial, or cervical lymph node lesions are the most common clinical presentations, and often, no ante-mortem clinical signs are apparent (Slany et al., 2016).

Differentiation of *M. avium* to the subspecies level in clinical practice is hindered by the need for specialized methods typically confined to research only. Common typing techniques include restriction fragment length polymorphism (RFLP) analysis utilizing various insertion sequences (IS; Mijs et al., 2002; Johansen et al., 2005; Moravkova et al., 2008; Rindi and Garzelli, 2014) or variable number tandem repeats (VNTRs) typing using mycobacterial interspersed repetitive units (MIRUs; Radomski et al., 2010; Rindi and Garzelli, 2014). Ambiguities can arise from these typing techniques as some IS elements share high sequence identity (Johansen et al., 2005) and VNTR–MIRU discrimination may not be sufficient to distinguish some isolates (Pate et al., 2011).

Evidence for the close relationship between MAA and MAS is abundant; however, the relationships between all subspecies have not been widely studied (Turenne et al., 2007; Paustian et al., 2008; Radomski et al., 2010). The true genetic diversity present within each subspecies of *M. avium* is another knowledge gap. Previous studies have focused on type strains (Bannantine et al., 2012; Möbius et al., 2015); a limited number of genes (Turenne et al., 2008) or a small number of isolates (Wibberg et al., 2020; Bannantine et al., 2020a,b). Microarray technology has revealed several large sequence polymorphisms between avian (MAA) and ruminant (MAP) isolates (Paustian et al., 2005, 2008). However, this technique uses a single reference strain to compare against other isolates. Limited conclusions can be drawn for isolates that were not directly compared to the reference strain. Furthermore, genomic regions that are absent from the reference strain but present in other isolates may not be recognized. A recent study utilized 29 closed genomes and discovered several genes that were subspecies-specific. However, this investigation was limited by a small dataset.

Whole-genome comparisons intuitively would allow accurate and comprehensive comparison of isolates; however, such methods can also be problematic for subspecies delimitation when a small number of isolates are used to describe taxa. The 70% DNA–DNA hybridization (DDH; Meier-Kolthoff et al., 2013) or 97% average nucleotide identity (ANI; Lee et al., 2016b) cut offs that have traditionally been used for species delimitation fail to distinguish between *M. avium* subspecies and other closely related MAC mycobacteria (Riojas et al., 2018; Tortoli et al., 2019). The concern with these methods is their reliance on a single strain type to represent a species. This can create complications in downstream analysis when intraspecies diversity makes it difficult to classify new isolates of unknown species. Furthermore, relative to many other bacterial genera, mycobacteria are a genetically homogenous group, yet they have diverse lifestyles and growth characteristics and exist in a broad range of niches. This indicates that the variability that does exist is biologically significant, and an accurate resolution of this variability is required. Consequently, DDH and ANI cut off values alone may not to be appropriate for the definition of mycobacterial species.

The understanding of *M. tuberculosis* diversity and lineages enables efficient outbreak tracing (Gardy et al., 2011) and informs epidemiologists to enable identification of the source of an outbreak and formulation of optimal control measures (Didelot et al., 2016). Arguably, identification of *M. avium* pathogens to the subspecies level is also crucial for understanding their significance and to perform epidemiological studies. However, relatively recent taxonomic studies concluded that the subspecies should be removed from *M. avium* taxonomy as the threshold for subspecies demarcation is not reached (Riojas et al., 2018; Tortoli et al., 2019). This conclusion was based on results from single type strains of MAP, MAA, and MAH. Regardless of the nomenclature, significant biological and phenotypic differences between the subspecies of *M. avium* were recognized by Thorel and Mijs (Thorel et al., 1990; Mijs et al., 2002) in niche adaptation, host preference, and growth characteristics (Figure 1). The availability of whole-genome sequencing (WGS) and the expansion of public genomic databases provide an opportunity to study many *M. avium* genomes and to make recommendations based on comprehensive analysis.

In this large-scale study, publicly available and 73 newly sequenced *M. avium* genomes were analyzed to compare subspecies clusters using pan-genome and SNPs analysis approaches, to identify subspecies-specific genes and mutation hotspots. A panel of subspecies-specific genes were identified that may serve as valuable biomarkers, and the SNP hotspot analysis demonstrated common regions between subspecies that have undergone extensive mutations during niche adaptation. Together, these outcomes will inform epidemiological analysis, lead to better disease control in animals and so reduce the chance of spillover into humans.

## Materials and Methods

### Isolate and Metadata Collation

Publicly available WGS data of *M. avium* isolates were sourced from the National Center for Biotechnology Information (NCBI) Sequence Read Archive (SRA) and the NCBI GenBank. Searches on public databases were undertaken on the 2/3/2021 for “*Mycobacterium avium*” and in the SRA database; filters for public, DNA, genome, paired, and Illumina were used. No raw reads were available from GenBank so assemblies were used. Any SRA isolate labelled as anything other than *Mycobacterium avium* or one of the subspecies (*silvaticum*, *paratuberculosis*, *avium*, or *hominissuis*) was removed from the dataset. Available public metadata are summarized in the excel spreadsheet in Supplementary Material 1. Additionally, 10 isolates were sequenced by the Mycobacterium Reference Laboratory, Westmead Hospital, NSW, 48 animal derived isolates were sequenced by the Farm Animal Health Group, University of Sydney, and 15 archival isolates of *M. avium* provided by the Mycobacterial Reference Laboratory Queensland were sequenced for this study. The archival isolates were recognized strains of the International Working Group on Mycobacterial Taxonomy (IWGMT; Wayne et al., 1993) and represented most serovars of *M. avium*: serovar 1 (Strains 17 and 1), serovar 2 (Strains 19, 55, and 60), serovar 3 (Strains 26 and 38), serovar 4 (Strains 54 and 62), serovar 5 (Strain 23), serovar 8 (Strain 29), serovar 9 (Strains 18 and 28), serovar 10 (Strain 49), and serovar 11 (Strain 31; Wayne et al., 1993). These serovar isolates were selected to ensure a set of representative serovars that were included in this investigation. Their details are summarized in the excel spreadsheet in Supplementary Material 1.

### Culture and DNA Extraction

To ensure that a high quantity and quality of DNA was extracted, a lengthy culture and DNA extraction procedure were undertaken for isolates sequenced by the University of Sydney as previously described (Mizzi et al., 2021). The process involved mechanical and enzymatic cell wall digestion, followed by a combination of hexadecyltrimethylammonium bromide/saline (CTAB/NaCl) and phenol–chloroform extraction.

### Quality Control and Assembly

**Figure 2:**
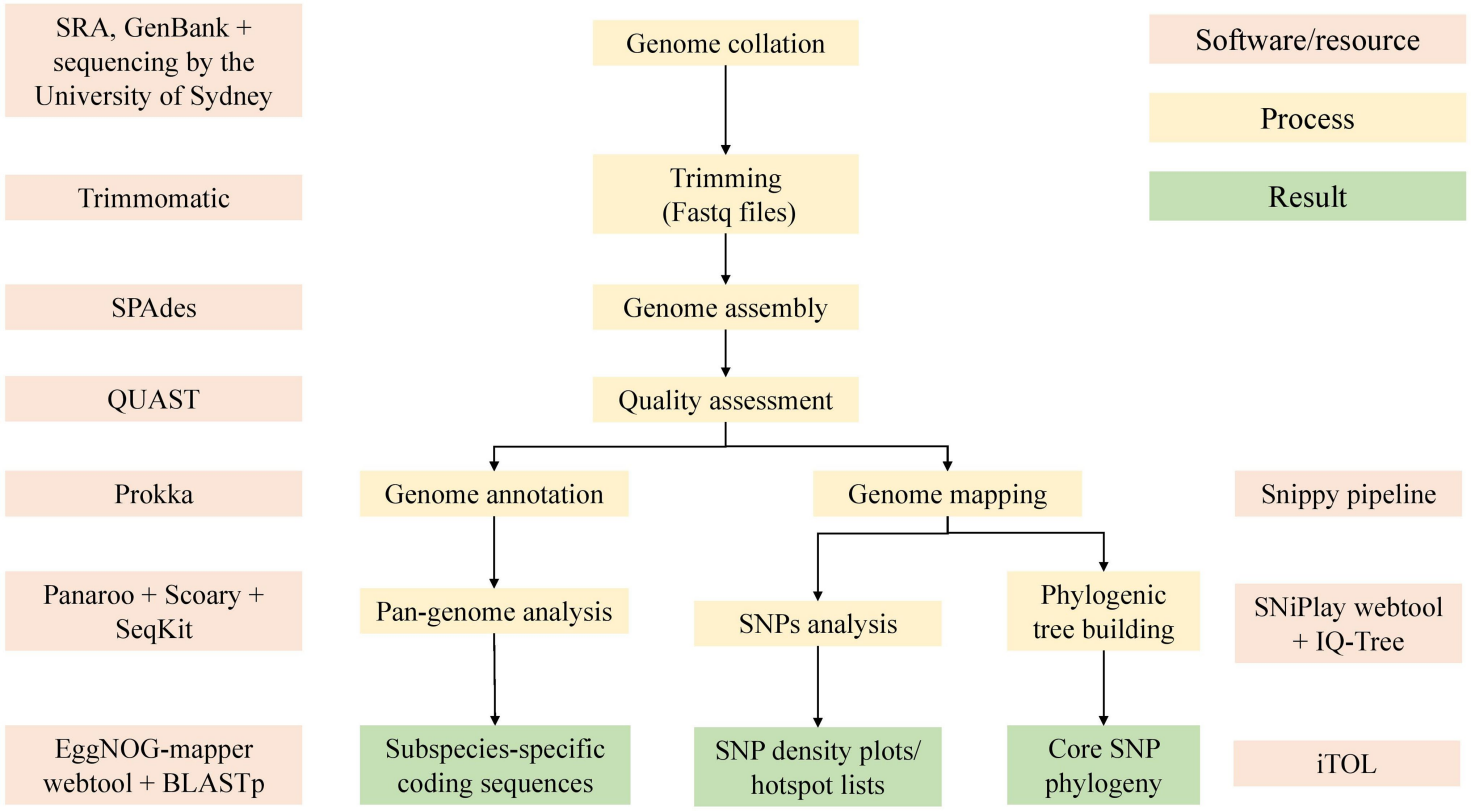
An overview of the bioinformatic methods used in this study. Software used included Trimmomatic (version 0.36; Bolger et al., 2014), SPAdes (Bankevich et al., 2012), QUAST (Gurevich et al., 2013), Prokka (Seemann, 2014), Panaroo (Tonkin-Hill et al., 2020), Snippy pipeline (Seemann, 2019), SNiPlay (Dereeper et al., 2011), EggNOG (Huerta-Cepas et al., 2017), IQ-Tree (Nguyen et al., 2015), and iTol (Letunic and Bork, 2019).

All fastq files were trimmed using Trimmomatic (version 0.36, RRID:SCR_011848; Bolger et al., 2014) with options set to -phred33, LEADING:3 TRAILING:3 SLIDINGWINDOW:4:20 MINLEN:36. Any genome with a g-zipped fastq file (forward, reverse, or both) with less than 50,000,000 bytes after trimming was discarded. Reads were assembled with SPAdes (version 3.12.0, RRID:SCR_000131; Bankevich et al., 2012) using the default k-mer size testing options. To improve the assemblies, the Bayes–Hammer read correction, and careful option for post-assembly Burrows Wheeler Aligner mismatch correction (Li and Durbin, 2009) were used. Quality assessment of the newly created assemblies and those obtained from GenBank were done with QUAST (version 5.0.2, RRID:SCR_001228; Gurevich et al., 2013). Assemblies with a GC% of less than 68%, number of contigs >500, or a total length outside of 4.5–6.2 megabases were removed from the study. A summary of the bioinformatics methods is depicted in Figure 2.

### Pan-Genome Analysis

Genome annotation was undertaken with Prokka (version 1.14.5, RRID:SCR_01473; Seemann, 2014) with the minimum contig length set to 500 base pairs. The Panaroo (version 1.2.3, RRID:SCR_021090; Tonkin-Hill et al., 2020) pan-genome pipeline was used for pan-genome analysis using the parameters for strict clean mode to remove contaminants and a sequence identity threshold of 0.75. Within the total number of genes identified by Panaroo software in the pan-genome analysis, there were several categories of genes distinguished by the number of isolates that contained a given gene. Core genes are those present in 99%–100% of isolates. The accessory genome, or portion not present in all isolates, is split into the soft core genes that are present in ≤95 to <99% of genomes, shell genes that are present in 15% ≤ to <95%, and cloud genes, which are present in <15% of strains.

### SNP Analysis

All genomes were mapped to one closed reference strain for each subspecies except for MAS as there was no closed reference genome available. These included the K10 strain for MAP, strain MAH104 for MAH and the Chester (DSM44156) strain for MAA. Summary statistics of the reference genomes is available in the supplementary tables (Supplementary Material 4). The Snippy pipeline (version 3.1; available at https://github.com/tseemann/snippy) was used with the default settings. Genome assemblies were used as the input given raw read files were not available for genomes downloaded from GenBank. The core.txt output file was viewed to identify the percentage of artificial reads mapping to the K10 reference genome. The core SNP alignment output file from Snippy was used by IQ-Tree to create a tree based on SNPs in the core genome. In the initial preliminary tree, the *Mycobacterium intracellulare* type strain ATCC13950 and *Mycobacterium intracellulare* subspecies chimaera type strain DSM44623 were added to the dataset. Any genomes that clustered with ATCC13950 or DSM44623 were removed from the study. The output using the MAP K10 reference genome was used as the final tree. Other tree outputs using the MAH and MAA reference genome are available in the supplementary figures (Supplementary Material 2).

To identify potential SNP hotspots in the subspecies, we assessed the SNP density throughout the dataset. This was done by using the location of high-quality core SNPs present in the combined VCF output from Snippy. This file was used as an input for the SNiPlay variant density viewer webtool (Dereeper et al., 2011). A sliding window of 10,000 bp was used in SNiPlay to produce SNP density plots. SNP hotspots were defined as a region that had a SNP density greater than four SDs from the mean SNP density across the reference genome. The 10,000 bp regions that displayed the highest number of SNPs from each dataset to each reference genome were extracted from the reference genome using bedtools. Hotspot regions were annotated with Prokka without the minimum contig length restriction, and the resulting protein fasta files were uploaded to the EggNOG functional annotation mapper webtool (version 2; Huerta-Cepas et al., 2017) with the taxonomic scope set to *Mycobacteriaceae* for further annotation.

### Phylogenetic Analyses

IQ-Tree (version 1.6.7, RRID:SCR_017254; Nguyen et al., 2015) was used to generate trees using the GTR + I + G model. Trees were visualized and annotated in iTol (RRID:SCR_018174; Letunic and Bork, 2019). Initially, the type strains for *Mycobacterium intracellulare* and *Mycobacterium chimaera* were included in the dataset to determine whether any genomes in the dataset were incorrectly labelled as *Mycobacterium avium*. Genomes that clustered with *M. intracellulare* or *M. chimaera* were very distant from majority of genomes in the study and removed from the dataset (*n* = 255, data not shown). Isolates were grouped into subspecies based on their clustering in the tree. Since only two typed isolates were available for the MAS subspecies, bird-type subspecies MAA and MAS were grouped together in this investigation and are referred to collectively as MAA/S. The phylogeny was rooted at the midpoint in iTol for ease of readability.

### Querying the Pan-Genome

Genes of interest for this study were those present in all isolates from one subspecies and absent in other subspecies. Genome-wide associations between genes identified in the pan-genome and subspecies were achieved with Scoary (RRID:SCR_021087; Brynildsrud et al., 2016) and validated with BLASTp. An association from Scoary was considered significant where a gene had a corrected value of *p* (Benjamini-Hochberg) of less than 0.05. Sequences of the genes of interest were extracted from the pan-genome reference output from Panaroo using seqkit (version 0.10.1; Shen et al., 2016). Coding sequences were converted from DNA sequences to protein sequences using Prokka. BLASTp was used to confirm the subspecies-specificity of each gene of interest. A gene was considered subspecies-specific if it had a matching identity of 97% or higher, *e*-value less than 0.000001, bitscore of 100, or greater and coverage of 95% of the full gene length or greater. Coding sequences of the markers identified by Scoary and validated by BLASTp were uploaded to EggNOG (available at http://eggnog-mapper.embl.de/, RRID:SCR_002456) with the taxonomic scope set to *Mycobacteriaceae*. To search for markers of pathogenicity, this approach was also used to compare the clonal pathogenic subspecies (MAA, MAS, and MAP) to the opportunistic pathogen MAH. A similar approach was taken to identify genes associated with respiratory or gastrointestinal tissue trophism, where MAP or not MAP was the trait of interest.

## Results

### Whole-Genome Sequencing and Assembly

Overall, the quality of published genomes and assemblies created from public raw data was highly variable. Many genomes that were accessible from the Sequence Read Archive (SRA) and GenBank were of undesirable quality and did not meet quality thresholds outline in the methods section (very high number of contigs, abnormally large or small file size or genome length, or GC% very low indicating contamination) for this investigation and thus were not included in the study.

At the time of searching (02/08/2021), 3,167 genomes were available on Sequence Read Archive (SRA) and 216 in GenBank. Of the total 3,383 available, 60 were removed as they were labeled as something other than *mycobacterium avium* such as *M. chimaera* or *M. intracellulare* in the metadata downloaded from the SRA. Post-trimming, 250 samples had one or more compressed Fastq files smaller than 50,000,000 bytes, 12 genomes failed to assemble, 226 samples had a GC% <68, 587 had more than 500 contigs, and 121 had a length outside of 4.5–5.3 megabases. Some samples did not meet multiple QUAST criteria.

During preliminary SNP analysis, the Snippy pipeline demonstrated that the MAH reference genome MAH104, MAA reference DSM44156 and MAP sheep strain Telford had 93.61, 93.78, and 98.9 percent of artificial reads map to the K10 MAP reference genome. Any sample that had less than 93% of artificial reads map to K10 was discarded (*n* = 624). An exception was made for the silvaticum reference genome, which had 87.25% of artificial reads map to K10. Once quality thresholds were met, an additional 194 genomes were removed as they were very distant in the phylogenetic tree (greater than 80,000 SNPs from K10) and clustered with ATCC13950 or DSM44623 (data not shown).

**Figure 3:**
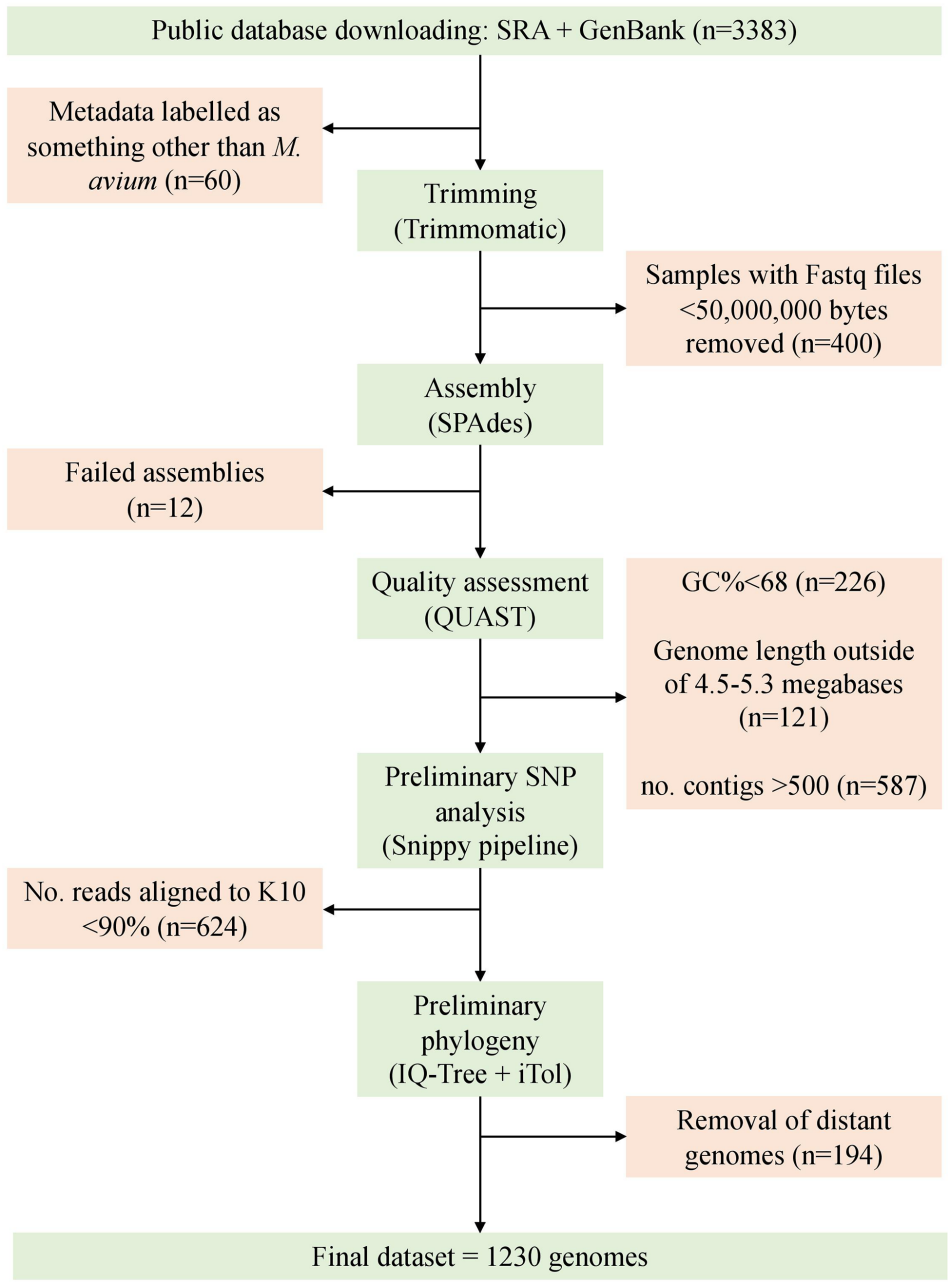
Overview of the process for sample selection and retention in this study. The final dataset consisted of 1,230 genomes.

The final dataset included 1,230 genomes that fell within the quality criteria (Figure 3). One exception was made for the single MAS-type strain isolate, which had 808 contigs and was only available from GenBank as an assembly. An assembly metrics summary table is provided within the supplementary tables, Supplementary Material 4. The number and broad characteristics of the *M. avium* WGS isolates included in the study are shown in Table 1.

**Table 1:** The reported host species, year range, and geographic origin, and the proportions of reported subspecies for 1,230 *Mycobacterium avium* genomes.

	MAP	MAA/S	MAH	Unreported subspecies	Total
Africa	2	0	0	0	2
Asia	10	2	60	0	72
America	400	0	72	106	578
Europe	111	3	45	132	291
Oceania	50	0	0	7	57
Not reported	n/a	n/a	n/a	230	230
Total	523	7	177	475	1,230
Host species	Cow (381), camel (2), sheep (70), goat (8), human (8), deer (3), environment (77), Bison (3), and Unknown (16)	Avian (4) and unknown (3)	Avian (2), cow (1), deer (2), environment (23), horse (1), human (149), pig (1), and unknown (3)	*Oryx dammah* (1), avian (46), environment (16), human (181), and unknown (228)	12
Year range	1975–2016	1901–2015	1983–2016	1995–2018	1901–2018
No. not reported	43	3	9	233	288

### Phylogeny

**Figure 4:**
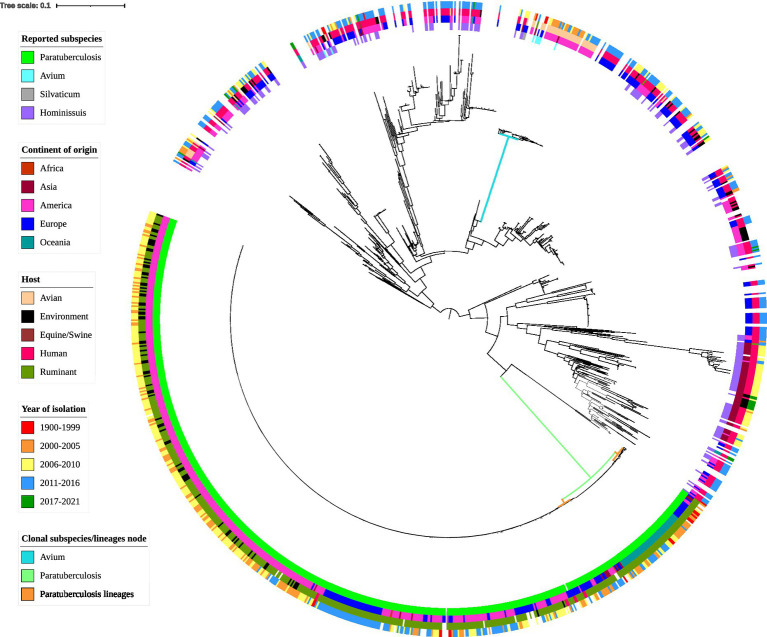
Whole-genome core SNP phylogenetic tree and associated metadata of 1,230 *M. avium* isolates based on *M. avium* subspecies *paratuberculosis* (MAP) K10 and rooted to the midpoint. Metadata are depicted by colored circles. Innermost circle is reported subspecies, second circle is the continent of origin of isolate, third circle is host species, and fourth circle is year of isolation. Isolates came from a variety of hosts; thus, some hosts such as avian (such as waterfowl and other poultry) and ruminant (such as sheep and cattle) species were combined within classes. For ease of readability, branch labels were removed. The green node and blue node indicate the MAP and *M. avium* subspecies *avium* (MAA)/S clusters. The smaller orange nodes indicate MAP lineages: the leftmost is the cattle lineage, the middle is the type I sheep lineage and the rightmost is the type III sheep lineage.

Whole-genome SNP phylogeny revealed tight clustering of certain subspecies (Figure 4). These tight clusters were present in the phylogeny regardless of which reference genome was used to produce the core SNP alignment and tree. The number of isolates in the MAP and MAA/S clades and the individual isolates present were identical between all three trees (supplementary figures, Supplementary Material 2). MAP K10 was used for the final tree as it is the most widely used reference genome. A tree produced with the Panaroo MAFFT alignment had similar branch positioning, but branch lengths were more variable within each subspecies.

MAP genomes formed a distinct clade (green node, Figure 4) and together the MAA and MAS subspecies formed another distinct clade (blue node, Figure 4). The two known MAS isolates formed a branch within the MAA cluster (see supplementary figures, Supplementary Material 2 for a more detailed MAA/S phylogeny). MAH was the most diverse of the subspecies in this analysis, with extensive branching and multiple clades present in the phylogenetic tree (Figure 4). Two isolates that had been previously typed as MAA, SRR8236370 (Operario et al., 2019) and SRR901356 [also known as *Mycobacterium avium* subsp. *avium* 2,285 (*R*)] are located outside of the MAA/S (MAA and MAS) cluster.

Within the MAP clade, there appeared to be two major lineages and within the smaller of these there appeared to be two sub-lineages: one contained genomes predominantly from the Oceania region, while the other had genomes originating from Europe and America (orange nodes, Figure 4). Genomes in the smaller major lineage contained sub-lineages predominantly from sheep and several have been typed as sheep strains of MAP. It is likely that this cluster represents the previously described sheep Type I and Type III sub-lineages of MAP. This is further supported by the presence of the Australian Telford Type I closed sheep genome in one sub-lineage and the S397 (American) and JIII-386 (German) Type III reference strains in the other sub-branch. The larger major lineage within the MAP clade contained isolates that were previously typed as cattle strains (or Type II) and are from predominantly bovine and human hosts. This larger branch within MAP contained the K10 Type II reference genome (see supplementary figures, Supplementary Material 2 for a more detailed MAP phylogeny).

### SNP Analysis

To identify the presence of SNP hotspots, defined as a 10,000 base-pair region where the number of core SNPs was four SDs greater than the average SNP density across the genome, the location of core genome SNPs was assessed for each subspecies group and then collectively across all *M. avium* genomes (Table 2). The reference genome used in the analysis impacted the identification of SNP hotspots. It should be noted that the number of core genome SNPs in the full dataset of 1,230 genomes (“All” category of Table 2) did not always equal the number of core genome SNPs detected in the subspecies group analysis, as the core genome differed between the collective *M. avium* group and each of the subspecies groups. This meant that any SNPs specific to the core of one subspecies may not be detected in the full dataset across the different subspecies.

**Table 2:** Number and density of SNPs and SNP hotpots for each subspecies and the full dataset, compared to the K10 (MAP), DSM44156 (MAA), and MAH104 (MAP) reference genomes.

Subspecies	Reference genome	Total SNPs	Average SNPs per 10,000 bp	Max	SD	No. hotspots	No. of regions with no SNPs
MAP	K10	88,814	20.6	49	7.5	39	11
	DSM44156	34,286	69.1	465	44.2	8	26
	MAH104	32,850	59.9	350	37.2	7	77
MAA/S	K10	88,814	97.5	673	59.6	10	16
	DSM44156	7,698	15.5	47	6.5	22	18
	MAH104	29,767	54.3	675	56.4	6	70
MAH	K10	91,519	189.5	655	116.1	13	44
	DSM44156	88,814	179.1	704	118.9	54	12
	MAH104	86,006	156.9	681	116.7	7	102
All*	K10	65,313	189.5	655	116.1	13	44
	DSM44156	65,049	131.1	497	92.1	9	69
	MAH104	65,206	118.9	459	92.7	8	119

* Full dataset comprising all 1,230 *M. avium* isolates.

When MAP isolates were mapped to the K10 MAP reference genome, the average core SNP density was 20.6. This was less than half of the average SNP density determined for the other subspecies when mapped to the K10 reference genome, with SNP densities of 97.5 and 189.5 per 10,000 base pairs for MAA/S and MAH, respectively. Similarly, when MAA/S isolates were mapped to the DSM44156 MAA reference genome, the average SNP density per 10,000 base pairs was 15.5, whereas against MAP and MAH isolates, it was 69.1 and 179.1, respectively. In contrast, when MAH isolates were mapped to the MAH104 reference genome, they had an average SNP density of 156.9. Against the MAH reference genome, MAP and MAA/S isolates had a lower density compared to the MAH subspecies at 59.9 and 54.3 SNPs per 10,000 base pairs, respectively.

### SNP Hotspots

In the full dataset, there were 13, 9, and 8 hotspots (where the number of SNPs present in a 10,000 base-pair region was greater than four standard deviations from the average SNP density) and 44, 69, and 119 regions where no SNPs were present when isolates were mapped to K10 (MAP reference genome), DSM44156 (MAA reference genome), and MAH104 (MAH reference genome), respectively (Table 2).

#### MAA/S Dataset

**Figure 5:**
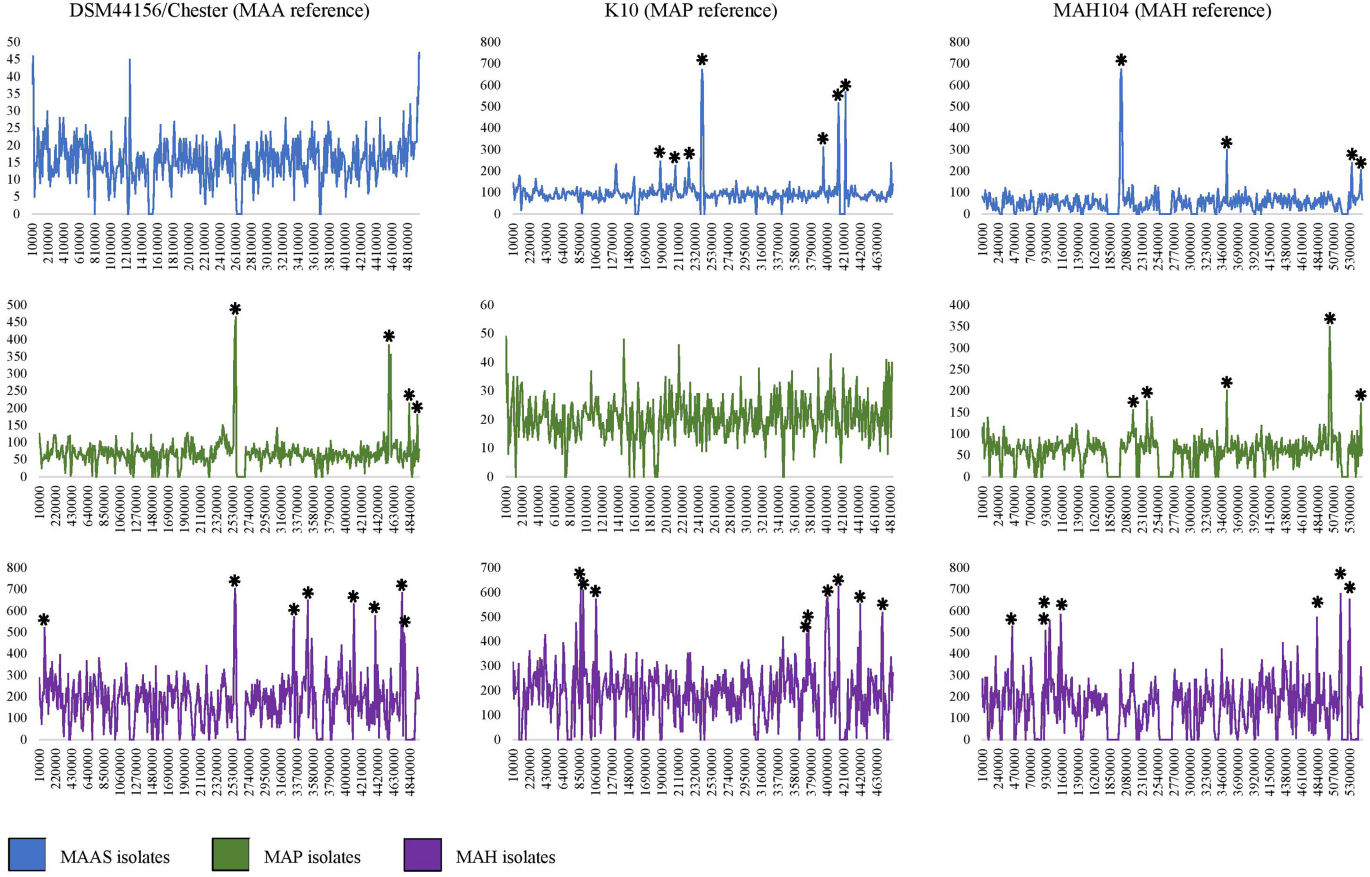
Density of SNP variants plots for MAP, MAA/S, *M. avium* subspecies *hominissuis* (MAH), and all isolates compared to closed reference genomes DSM44156 (MAA), K10 (MAP), and MAH104 (MAH). Several regions in each subspecies dataset can be clearly seen to contain several 100 SNPs. Significant peaks are indicated by an asterisk (*). Note where hotspots occurred over consecutive 10,000 bp ranges a single asterisk is used. The *Y* axis indicates the number of SNPs in a 10,000-bp region; the *X* axis is the position in the reference genome starting from the same recognized starting point (*dnaA*).

SNP analysis demonstrated the presence of several hotspot regions in all subspecies. The most notable were the hotspots in the MAA/S dataset (Figure 5). Several major hotspots were seen in these isolates compared to the K10 and MAH104 reference genomes. When the MAA/S dataset was mapped to the K10 reference genome, the largest peaks of 673 and 601 SNPs occurred in reference genome regions 2,410,000–2,420,000 and 2,420,000–2,430,000 base pairs. Within these two 10,000 base-pair regions in K10 are four genes belonging to the mycobactin (mbt) synthesis cluster and several enzymes involved in metabolism. The remainder of the significant peaks occurred at: 1,880,000; 2,240,000; 2,400,000; 3,950,000; 4,140,000; 4,150,000; and 4,240,000. The largest hotspot identified within the MAA/S dataset when compared to the MAH104 reference genome occurred over three 10,000-bp regions between 2,000,000 and 2,030,000 base pairs and contained a total of 1,890 SNPs. This region contained four genes from the mbt cluster, six genes involved in information storage and processing and a further six genes belonging to various Clusters of Orthologous Groups (COG) pathways. Other significant hotspots occurred at 3,530,000 and 5,330,000 bp and contained mainly hypothetical proteins.

#### MAP Dataset

Within the MAP dataset, the largest hotspots occurred when compared to the MAA reference genome, DSM44156; these were across two 10,000-bp regions of the reference genome between 2,560,000 and 2,580,000 bp and contained 899 SNPs (Figure 5). In these regions of the DSM44156 genome, there are four mbt genes, four genes involved in metabolism, three in transcription and three genes were poorly characterized. Other hotspots in the MAP dataset when compared to the MAA reference genome occurred at: 2,550,000; 4,570,000; 4,580,000; 4,590,000; 4,830,000; and 4,940,000. The largest hotspot for the MAP dataset in comparison with the MAH104 reference genome comprised of 626 SNPs and occurred at base pairs 5,010,000–5,030,000 bp. In this section of the MAH reference genome, there were five genes from various metabolic pathways, four genes involved in transcription and its regulation, two genes involved in cell signaling, and four genes with poor characterization. No other hotspots in the MAP dataset compared to the MAH104 reference exceeded 250 SNPs per 10,000 base pairs; however, some less notable but still significant hotspots occurred at: 2,180,000; 2,380,000; 3,530,000; and 5,460,000.

#### MAH Dataset

The MAH dataset had the greatest number of significant peaks in the SNP variant plots and had the highest SNP density compared to all of the reference genomes (Table 2; Figure 5). When compared to the K10 reference genome, there were 13 significant peak regions ranging from 471 to 655 SNPs per 10,000 base pairs. These peaks were spread throughout the K10 genome and contained genes belonging to a variety of functional categories. The exact locations were at base pairs: 810,000; 870,000; 900,000; 1,060,000; 1,070,000; 3,760,000; 3,980,000; 3,990,000; 4,000,000; 4,010,000; 4,140,000; 4,420,000; and 4,700,000 within K10. For readability, a single asterisk is used where hotspots are located in consecutive 10,000 bp segments (*n* = 4). Ten genes were associated with cellular processes and signaling COGs, 20 were associated with transcription, translation, and replication pathways, 38 genes had unknown functions, and 54 were involved in metabolism. Within the genes involved in metabolic processes, 18 genes were predicted to be involved in lipid transport and metabolism, while 14 genes were predicted to have an involvement in secondary metabolite biosynthesis, transport, and catabolism. When compared to the DSM44156 reference genome, the MAH dataset contained 12 significant peaks with 478–704 SNPs per 10,000 bp. These hotspot regions in the MAA reference genome contained 14 genes involved in cellular signaling pathways, 17 involved in information storage and processing, 47 from metabolic pathways, and 33 that were poorly characterized. The largest peak occurred over a region spanning 20,000 bp between 2,560,000 and 2,590,000, with a total of 1,309 SNPs identified. This is the same hotspot region that was identified in the MAP dataset when compared to the MAA reference genome. Other significant peaks occurred at: 80,000; 3,320,000; 3,330,000; 3,510,000; 4,110,000; 4,390,000; 4,730,000; 4,740,000; 4,760,000; and 4,770,000 bp.

*Mycobacterium avium* subspecies *hominissuis* was unique in that it was the only subspecies to exhibit significant peaks when compared to a reference genome that belonged to the same subspecies. Eight significant peaks were found in the MAH dataset when compared to the MAH104 reference genome. These occurred throughout the genome and ranged from 466 to 681 SNPs per 10,000 base pairs. The location in MAH104 with the most SNPs occurred at 5,170,000 base pairs. Other hotspots occurred at: 440,000; 920,000; 990,000; 1,140,000; 4,830,000; and 5,300,000 bp. Prokka annotated nine genes within this region and EggNOG identified two associated with metabolism, three in information storage and processing, one associated with cellular signaling, and two poorly characterized genes.

### Pan-Genome Analysis

**Figure 6:**
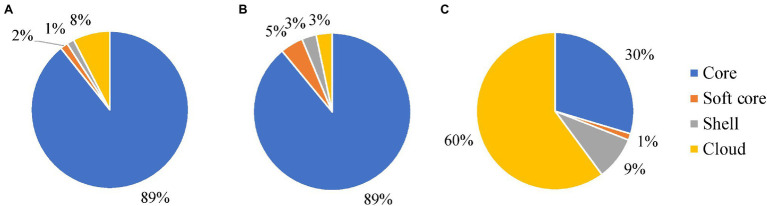
The relative proportions of genes belonging to the core, soft core, shell, and cloud genomes of MAP **(A)**, MAA/S **(B)**, and MAH **(C)** datasets. A table containing the number of genes in each section is available in the supplementary tables, Supplementary Material 4.

*Mycobacterium avium* subspecies *hominissuis* had the smallest number of core genes and the largest number of accessory and cloud genes of all the *M. avium* subspecies (Figure 6). There were: 4,055; 182; 1,220; and 8,227 genes in the core, soft core, shell, and cloud and a total number of 13,684 genes. The MAA/S group had a total of 4,688 genes and a breakdown of: 4,174; 220; 140; and 154 genes in the core, soft core, shell, and cloud, respectively. Despite having more than 10 times the number of isolates in the dataset compared to MAA/S and only 31 fewer isolates than MAH, the MAP subspecies had the largest core genome and the smallest number of accessory genes. This indicates a much lower diversity within this subspecies. MAP had a total of 4,777 genes with a core of 4,267 and an accessory genome 75, 72, and 363 genes in the soft core, shell, and cloud. Overall, the proportion of genes in each category was similar in MAA/S and MAP, where the core genome made up 89% of the total number of genes. In contrast, MAH had only 30% of genes that were classified as being in the core and the majority of genes (60%) were within the cloud genome (Figure 6 and supplementary tables, Supplementary Material 4).

### Subspecies-Specific Marker Genes

**Figure 7:**
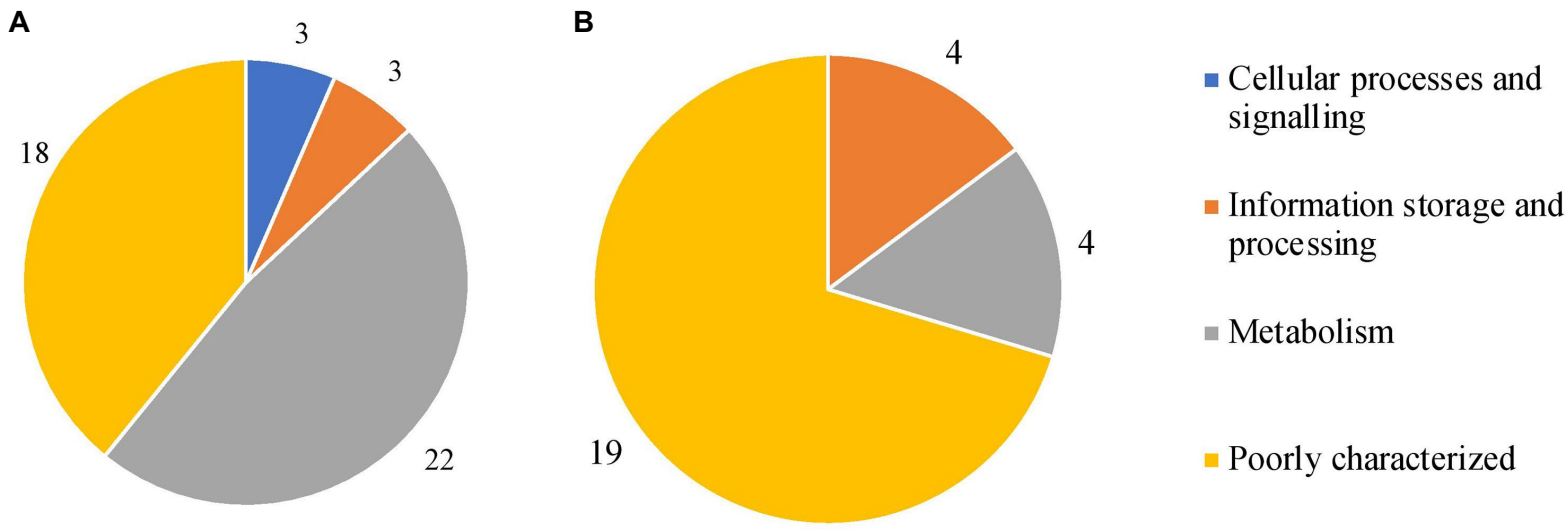
Clusters of Orthologous Groups (COG) categories for all marker genes in MAP **(A)** and MAA/S **(B)**. These genes were present in all whole-genome sequencing (WGS) isolates from their respective subspecies (MAP *n* = 575, MAA/S *n* = 49) and absent from all other subspecies.

Scoary software identified 76, 37 and three subspecies-specific markers for MAP, MAA/S, and MAH, respectively. Of these, 46, 27, and two genes for MAP, MAA/S, and MAH reached the BLAST thresholds of a matching identity greater than or equal to 97%, *e*-value less than 0.000001, bitscore of 100, or greater and coverage of 95% of the full gene length or higher. EggNOG software demonstrated that these marker genes came from a variety of functional categories. Of the genes that had been validated with BLAST, 37 and 13 genes from MAP and MAA/S and the two from MAH were successfully functionally annotated by EggNOG (Figure 7, supplementary data, Supplementary Material 3).

In MAP, most of the subspecies-specific marker genes belonged to the metabolism COG category. Specifically, 15 MAP-specific genes were on COG pathways that are involved in inorganic ion and secondary metabolite biosynthesis, transport, and catabolism (categories P and Q; Galperin et al., 2021). The majority of the markers found in MAA/S were of unknown function (19 of 27); the genes that were known were associated with metabolism or information storage and processing (Figure 7). Both of the two species-specific markers in MAH were involved in metabolic pathways. One of the genes, a fumarylacetoacetate (FAA) hydrolase, belonged specifically to category Q; secondary metabolites biosynthesis, transport, and catabolism pathways. The other gene was a glyoxalase bleomycin resistance protein dioxygenase (bphC_1) and belonged to the amino acid transport and metabolism (category E).

## Discussion

This investigation aimed to describe genetic differences between the *M. avium* subspecies using a pan-genome and mapping approach. The dataset included 1,230 genomes from a variety of geographical locations, host species, and years. This included 21 isolates sequenced specifically for this investigation and deposited in the SRA. To our knowledge, this dataset represents the largest number of *M. avium* genomes that has been used for comparative genomics of these subspecies. Furthermore, the large phylogeny that this study provides may be useful for outbreak tracing, should an *M. avium* outbreak occur. The novel SNP hotspot approach that was undertaken enabled the identification of regions that have undergone extensive mutation during niche adaptation. Subspecies-specific putative virulence genes based on homologies to genes with similar functions to other taxa were identified by the pan-genome analysis. The core SNP phylogeny revealed tight clustering of MAP and MAA/S subspecies and high diversity of MAH. The phenomena of niche adaptation by these subspecies appears to have occurred by a combination of mutations within regions common to all subspecies and the loss or gain of additional genes required for survival in their respective environments.

Phylogenomic analysis demonstrated a clear distinction between subspecies. Many of these isolates had been described only to the species level and had not been subtyped, but all known MAA/S and MAP subspecies clustered together. MAA and MAS formed one tight clade while MAP formed another, separate, tight clade. Two isolates that had been previously typed as MAA, SRR8236370 (Operario et al., 2019) and SRR901356 [also known as *Mycobacterium avium* subsp. *avium* 2,285 (*R*)] were located outside of the MAA/S (MAA and MAS) cluster, suggesting that they had been misclassified originally. This is supported by previous investigation where 2285R was seen within a clade of MAH genomes (Uchiya et al., 2013). MAH demonstrated high genomic diversity and did not form a single clade. Known MAH isolates were scattered throughout the phylogeny but were absent from the MAP and MAA/S branches. An earlier study suggested that MAA/MAS and MAP are pathogenic clones within *M. avium* (Turenne et al., 2008). This is supported by the phylogeny of the present study, where the MAP and MAA/S appeared to be sub-clades within MAH. MAP is considered the most clonal of the subspecies with MAA intermediate but much less diverse than MAH (Kei-Ichi et al., 2017). Other investigators also reported clustering of subspecies (Kei-Ichi et al., 2017; Bannantine et al., 2020a). When MAP and non-MAP genomes underwent phylogenomic analysis, MAA strains were intermediate to MAH and MAP strains and two distinct clusters, one MAP and one non-MAP were present (Bannantine et al., 2020a). A slightly larger dataset demonstrated three distinct clusters, one consisting of MAH isolates, another of MAA and the MAS-type strain and a final cluster split into two sub-lineages with MAP isolates contained within one sub-lineage and two MAH isolates (A5 and 10-4249) in the remaining sub-lineage (Kei-Ichi et al., 2017). The two MAH isolates (A5 and 10-4249) that were close to MAP in Kei-Ichi and colleagues’ investigation were also situated close to MAP isolates in the present study along with several other MAH genomes.

The midpoint root in the phylogeny resulted in two distinct branches of *M. avium*. One contained roughly half of the MAH isolates and the MAA/S cluster and the other contained the remaining MAH isolates and the MAP cluster. Suggestions to subdivide MAH into multiple subspecies have been put forward (Turenne et al., 2008), and the results of the present study support this proposal. Identification of specific lineages associated with virulent genotypes or host tropism provides an opportunity to understand how pathogens evolve. Genomic differences between lineages of MAP have been studied extensively. Numerous investigations implicate lineage-specific regions and mutations as a reason for phenotypic differences between strains (Bannantine et al., 2012; Wibberg et al., 2020; Lim et al., 2021). Knowledge of strain-specific characteristics that are linked with virulence or host tropism is crucial for understanding pathogenesis. Applying similar methods to lineages of MAH within the current dataset may provide valuable insights into how MAH is able to cause disease in some instances. Further work to investigate differences between MAA-like and MAP-like MAH isolates separated by the midpoint may reveal novel insights on pathogenicity and host-specificity for these groups and provide phenotypic support for the split of MAH. This may reveal gene or SNP associations with certain phenotypes or lineages. Such investigations on a smaller scale have been undertaken. Several loci specific to the hypervirulent TH135 MAH reference genome were associated with isolates from patients with progressive pulmonary disease (Kei-Ichi et al., 2017). The authors suggest these distinct features may have been acquired during strain evolution and could play an important role in the progression of clinical MAC disease. Distinct genetic features have been found between isolates that display increased virulence or progressive disease in immunocompetent patients (Uchiya et al., 2013) including plasmids (Takahashi et al., 2015) and specific lineages (Kei-Ichi et al., 2017). Further *in vitro* work may assist in improving our understanding of virulent genotypes in various regions and countries. Knowledge in this area may improve patient prognosis and treatment outcomes.

The SNP and pan-genome analyses told a similar story, with MAH considered the most diverse subspecies and MAA/S and MAP considerably less diverse. The largest accessory genome and smallest core genome (30%) were found in MAH isolates. In contrast, pathogenic subspecies MAP and MAA/S have a large core (89%) and comparatively smaller accessory component. Similar pan-genome findings were reported previously for MAP and MAH (Bannantine et al., 2020a,b). The inherent diversity between MAH isolates was also evident in the mutation hotspot analysis, with multiple SNP hotspots identified when the MAH dataset was compared to a MAH reference genome. In contrast, no significant hotspots were seen in MAP and MAA/S datasets when mapped to a reference genome of their own subspecies, indicative of a higher clonality within these subspecies. Further, MAH had a high number of SNPs compared to all reference genomes. The high level of diversity within the MAH subspecies and the very limited diversity of MAP, MAA, and MAS has been demonstrated in other studies (Turenne et al., 2008; Pate et al., 2011; Kei-Ichi et al., 2017; Bannantine et al., 2020a) and may reflect the saprophytic lifestyle of MAH; it is commonly found in the environment and likely to be subject to a wide range of conditions. In contrast, obligate pathogens, such as MAA, MAS, and MAP are exposed to a narrow range of conditions due to an intracellular lifestyle and are adapted to that environment. Thus, a high level of genomic diversity may not be necessary for survival of MAA, MAS, and MAP.

The present study found many subspecies-specific coding sequences in the MAA/S and MAP subspecies that may play a role in niche adaptation. Comparisons between the pathogenic subspecies (MAA, MAS, and MAP) and MAH revealed no common genes present in the clonal, pathogenic species that were absent in MAH. This may reflect the phenotype of gut infection in MAP vs. lung infection in MAA/S requiring different sets of virulence genes for successful infection in these tissues. Other studies have looked at subspecies-specific loci, and several genes have been found that match between studies. A group in Japan utilized 79 *M. avium* genomes and found several subspecies-specific loci containing virulence genes (Kei-Ichi et al., 2017). MAP and MAA/S were missing one locus that contains PPE proteins. A MAP-specific locus containing several coding sequences for genes encoding Mec, MmpL/MmpS, and PPE proteins was also discovered (Kei-Ichi et al., 2017). MAH-specific PPE proteins and MAP-specific Mec, MmpL/MmpS were not found in the present study; however, a MAP-specific PPE protein was identified. More recently, Bannantine and colleagues used a pan-genome approach on 29 closed *M. avium* genomes. They reported 86 genes specific to MAP, seven specific to MAA, and three that were specific to MAH (Bannantine et al., 2020b). Subspecies-specific loci and genes are likely to be associated with adaptation to human and porcine hosts by MAH or ruminant hosts by MAP.

Subspecies-specific markers found in the MAA/S subspecies were predominantly hypothetical proteins. The coding sequences that were successfully annotated were involved in metabolism, specifically energy production/conversion and transcription. Metabolic pathways were also represented in the SNPs analysis, with hotspots in the MAA/S dataset consistently in regions of the reference genomes that contained genes encoding enzymes involved in metabolism and mycobactin (mbt) synthesis. Previous work on metabolic pathways in MAA has demonstrated a requirement of cholesterol for virulence (De Chastellier and Thilo, 2006). The utilization of cholesterol and fatty acids has also been described in MAP (Weigoldt et al., 2013; Thirunavukkarasu et al., 2014), but no direct comparison of these two subspecies has been undertaken. Findings from a smaller, closed genome dataset revealed seven genes specific to MAA; several of these genes overlap with MAA-specific genes found in the present study. Future studies to uncover the function of the MAA/S-specific genes may reveal unique pathways that confer a survival advantage in avian hosts.

Within the SNP analysis, several genes from the mbt cluster were found in hotspot regions. In the MAA/S dataset, hotspots were found to the K10 (MAP) and MAH104 (MAH) reference genomes within regions that contained mbt cluster genes. The same mbt cluster genes were also found in hotspots within the MAP and MAH datasets when they were compared to the DSM44156 MAA reference genome. Studies in *M. avium* and other mycobacteria have demonstrated the importance of mycobactin and iron utilizing pathways for survival and virulence (De Voss et al., 1999; Fang et al., 2015). MAA and MAS may have adaptations in iron sequestering pathways that offer a survival advantage in their preferred avian hosts. Further *in vitro* experiments may be needed to fully appreciate the consequences of mutations present in these genes.

The largest subcategory of MAP-specific genes in the present study was associated with secondary metabolites biosynthesis, transport, and catabolism. A similar finding was also reported by another recent investigation (Bannantine et al., 2020b). Several genes were also annotated as mammalian cell entry (Mce) genes. Mce genes are involved in invasion and persistence within host cells (Chitale et al., 2001; El-Shazly et al., 2007; Chandolia et al., 2014). Interactions with host cell receptors have been described (Zhang et al., 2018) and may reflect the unique host and tissue tropisms of this subspecies. MAP has been the most extensively studied in the search for subspecies-specific genes (Gold et al., 2001; Rodriguez et al., 2002; Li et al., 2005; Janagama et al., 2009; Wang et al., 2014, 2016). Earlier investigations found some of the MAP-specific genes that were identified in the present study including a cytochrome P450, polyketide synthase, and several other genes predominantly involved in iron acquisition and metabolism. MAP is mycobactin dependant in culture conditions and has evolved MAP-specific iron sequestering pathways (Barclay et al., 1985; Collins et al., 1985). No MAP-specific genes that were annotated as mbt genes were identified in the present study. However, the large number of genes involved in secondary metabolites biosynthesis indicates there may be MAP-specific genes involved in this pathway. Further work interrogating these MAP-specific genes *in vitro* is required to reveal their functions to see if they are indeed linked to iron-sequestering pathways. Furthermore, these genes may be essential for the unique gut tropism of this subspecies, and additional study may reveal its virulence mechanisms.

Genes or SNPs that are specific to a particular subspecies may be suitable for use as diagnostic markers. Current methods used to differentiate *M. avium* subspecies are typically based on RFLP patterns. Various insertion sequences (IS) are exploited for this purpose including IS*1245* and IS*1311* for MAH (Mijs et al., 2002), IS*901* in MAA and MAS (absent from MAH) or the MAP-specific IS*900* (Moravkova et al., 2008; Rindi and Garzelli, 2014). SNPs within these IS elements can also be used to differentiate lineages within certain subspecies, such as IS*1311* in MAP (Whittington et al., 1998). IS*1311* shares 85% sequence identity with IS*1245* (Johansen et al., 2005), and IS*900* has apparent homologues in other mycobacterial species (Cousins et al., 1999). Variable number tandem repeat (VNTRs) typing using MIRUs has been developed and has higher resolution than PCRs based on insertion elements (Radomski et al., 2010; Rindi and Garzelli, 2014). However, sometimes this technique is insufficient for closely related isolates and a combination of techniques is required to distinguish between bird-type isolates (Pate et al., 2011). Use of whole genes for diagnostic tests may be more accurate than using insertion sequences or SNPs in particular genes. The recent publication by Bannantine and colleagues (Bannantine et al., 2020b) and the complementary results from the present investigation demonstrate there are a variety of subspecies-specific genes that could be used diagnostic targets. Further work would be required to determine the suitability of these marker genes in a larger dataset as candidates for diagnostic targets.

The main limitations of the study include use of *de novo* assemblies, culling of genomes that did not meet assembly standards (~30% of assemblies), strict cutoffs to call marker genes and a small number of MAA and MAS genomes. These limitations may have resulted in reduced apparent genomic diversity within the MAA and MAS genomes and fewer features relevant to MAA and MAS subspecies. For example, the inclusion of more MAS genomes may have revealed another distinct cluster within the MAA subspecies. Further, an absence of good coverage across some areas of genomes due to *de novo* assembly and strict criteria for marker genes to be called means that other subspecies-specific genes and loci likely exist. However, the thresholds that were used in this study minimized the chance of a gene marker being the result of an assembly error and improved the validity of the pan-genome results. Mycobacterial isolates are notoriously difficult to culture due to a slow growth rated compared to other bacterial species. Their sequencing and assembly is also difficult due to the presence of repetitive regions of high GC content. To overcome assembly and annotation artifacts, other researchers limited their study to closed genomes only (Bannantine et al., 2020a), an approach that would accurately demonstrate that the larger number of genes in the accessory genome of non-MAP subspecies is not due to assembly artifact but rather is a true reflection of the differences in diversity between MAH and other *M. avium* subspecies, which validates the pan-genome findings of the present study.

The use of a reference genome that belonged to a single subspecies for use in the final analysis may have introduced some bias within the phylogeny. Future *M. avium* studies should consider the use of a bioinformatically created most recent common ancestor (MRCA) as has been undertaken for the Mycobacterium tuberculosis complex (Comas et al., 2013). This method may reveal insights in the historic spread of *M. avium* globally and aid in controlling future transmission.

## Conclusion

Increasing our understanding of the genome of *M. avium* subspecies will lead to insights into mycobacterial virulence, pathogen evolution, host preference, and tissue tropisms in pathogenesis. This new information is vital for understanding the clinical significance of the MAC for human and animal health and will lead to improvements in diagnosis, control, and treatment. We confirm earlier findings from Turenne et al. (2008) that *Mycobacterium avium* is a species made up of one highly diverse subspecies, known as MAH, and at least two pathogenic sub-clones, namely MAA and MAP, that have adapted to specific host niches. Due to the small number of MAS isolates available, no conclusions could be drawn for this subspecies except that it is closely related to MAA. The mapping approach revealed several areas in each subspecies where extensive mutations have occurred relative to a reference genome from other subspecies. Hotspots occurred in regions where known mycobacterial virulence genes were present in the reference genome. The pan-genome analysis confirmed that MAH is highly diverse, whereas MAA/S and MAP are quite clonal, with the MAP subspecies being the most clonal. Several subspecies-specific coding sequences were found that belong to a variety of COG categories. These differences between subspecies may reflect their adaptation to different lifestyles.

## Data Availability Statement

The whole genome data generatated for this study can be found in the Sequence Read Archive https://www.ncbi.nlm.nih.gov/sra under BioProject ID PRJNA809746.

## Author Contributions

RM was responsible for the study design, dataset curation, bioinformatics analysis, and writing of the manuscript. VT and KP contributed to the study design and editing of the manuscript. RW assisted in the curation and description of genomes sequenced by the University of Sydney and editing of the manuscript. All authors contributed to the article and approved the submitted version.

## Funding

This work was supported by Meat and Livestock Australia (MLA; grant number P.PSH.0813). The funding body (MLA) provided support in the form of some authors’ salaries and research materials but did not have any role in the study design, data collection, analysis, or preparation of the manuscript.

## Conflict of Interest

The authors declare that the research was conducted in the absence of any commercial or financial relationships that could be construed as a potential conflict of interest.

## Publisher’s Note

All claims expressed in this article are solely those of the authors and do not necessarily represent those of their affiliated organizations, or those of the publisher, the editors and the reviewers. Any product that may be evaluated in this article, or claim that may be made by its manufacturer, is not guaranteed or endorsed by the publisher.
