# Supplementary material for: Global Phylogeny of Mycobacterium avium and Identification of Mutation Hotspots During Niche Adaptation
Source: Front Microbiol. 2022 May 6;13:892333. doi: 10.3389/fmicb.2022.892333 (PMC9121174; doi:10.3389/fmicb.2022.892333)
Supplement: Supplementary file 3 [file Data_Sheet_1.docx]

Supplementary table 1. Reference genomes used in this study

|  | **K10** | **DSM44156** | **MAH104** |
| --- | --- | --- | --- |
| **Subspecies** | MAP | MAA | MAH |
| **Length** | 4,829,781 | 4,956,929 | 5,475,491 |
| **GC%** | 69.3 | 69.3 | 69.0 |
| **Year** | Mid 1970s | 1901 | Mid 1980s |
| **Location** | USA | Europe | USA |
| **Host** | Cow | Hen | Human |
| **Reference** | Li et al. (2005a) | Goethe et al. (2020) | Kathleen et al. (2006) |

Supplementary table 2. Assembly metrics summary. Note, these metrics were calculated only on assemblies which met quality requirements (GC% greater than 68%, number of contigs <500 and total length 4.5-6.2 megabases). All values are to one decimal place. Statistics for contigs do not include the complete genomes or the MAS type strain ATCC49884.

|  | **All** | ***M. paratuberculosis*** | ***M. avium/ silvaticum*** | ***M. hominissuis*** |
| --- | --- | --- | --- | --- |
| **No. genomes** | 1230 | 575 | 49 | 606 |
| **No. complete genomes** | 28 | 13 | 3 | 12 |
| **GC%** | 69.1 | 69.3 | 69.29 | 68.99 |
| **Genome length (bp)** | 5048113.7 | 4795599.9 | 4877595.5 | 5301487 |
| **No. contigs** | 180.5 | 173.9 | 121.4 | 190.2 |

Supplementary table. 3 Number of genes present in the core, soft core, accessory and cloud genome of the subspecies and total dataset. Note that the number of genes in each group does not add up in the ‘All’ section as Panaroo was run separately on each subspecies.

|  | **MAP** | **MAA/S** | **MAH** | **All** |
| --- | --- | --- | --- | --- |
| **Total** | 4777 | 4688 | 13684 | 13959 |
| **Core**  (99% < strains ≤ 100%) | 4267 | 4174 | 4055 | 3942 |
| **Soft core**  (95% < strains ≤ 99%) | 75 | 220 | 182 | 132 |
| **Shell**  (15% < strains ≤ 95%) | 72 | 140 | 1220 | 849 |
| **Cloud**  (0% < strains ≤ 15%) | 363 | 154 | 8227 | 9036 |
