# Supplementary material for: Global Phylogeny of Mycobacterium avium and Identification of Mutation Hotspots During Niche Adaptation
Source: Front Microbiol. 2022 May 6;13:892333. doi: 10.3389/fmicb.2022.892333 (PMC9121174; doi:10.3389/fmicb.2022.892333)
Supplement: Supplementary file 4 [file Data_Sheet_1.PDF]

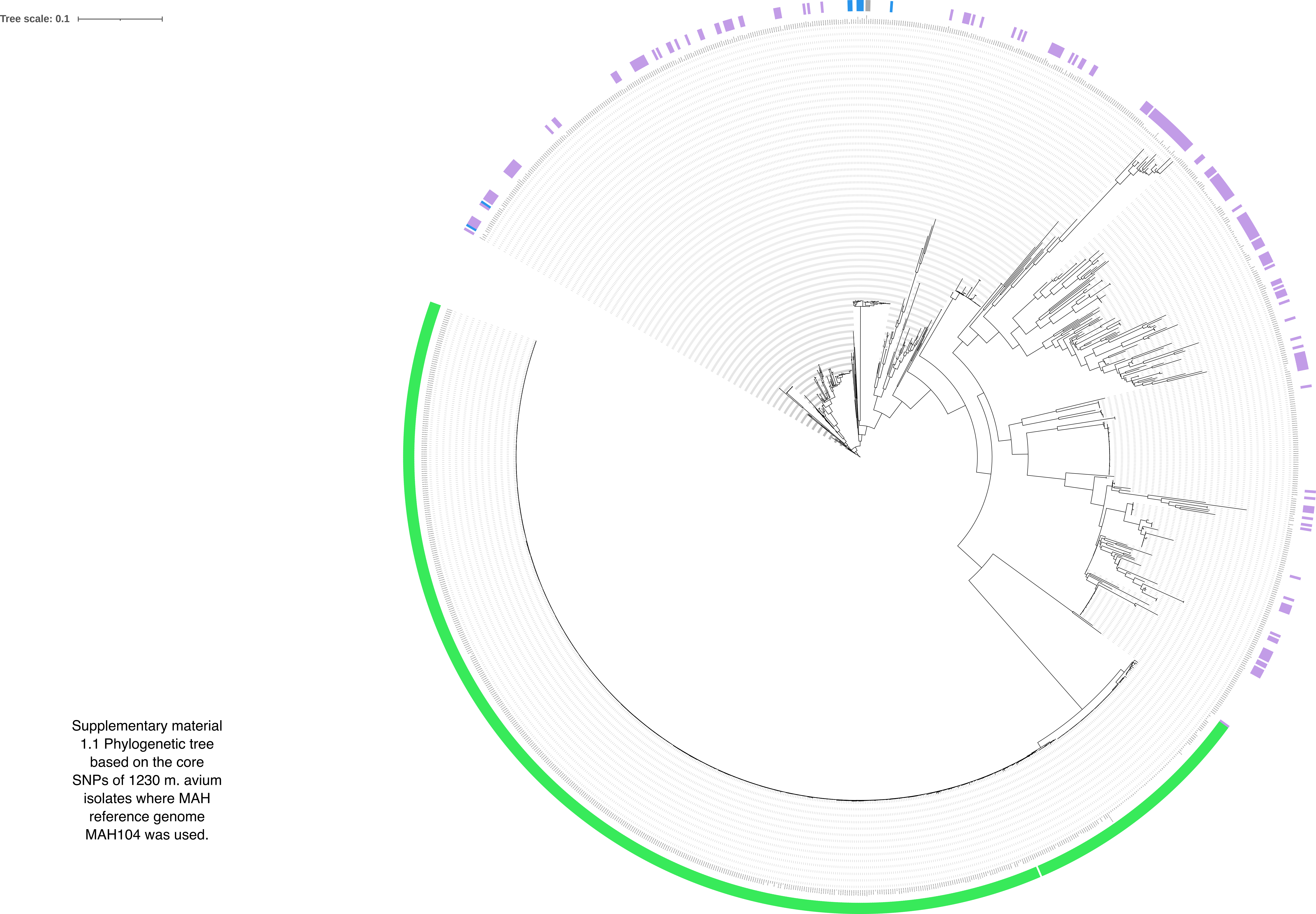

Supplementary material 1.1 Phylogenetic tree based on the core SNPs of 1230 m. avium isolates where MAH reference genome MAH104 was used.

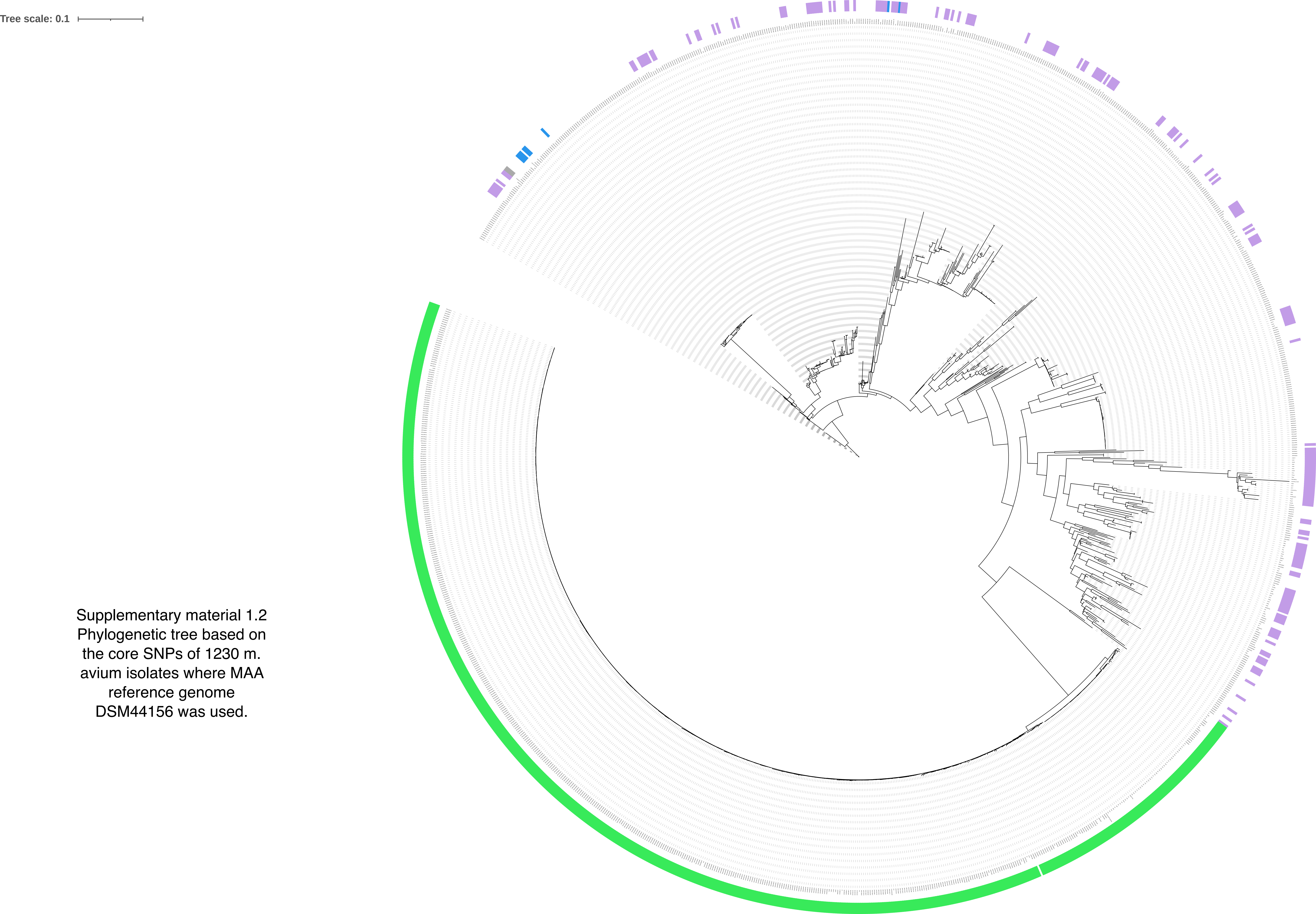

Supplementary material 1.2
Phylogenetic tree based on the core SNPs of 1230 m. avium isolates where MAA reference genome DSM44156 was used.

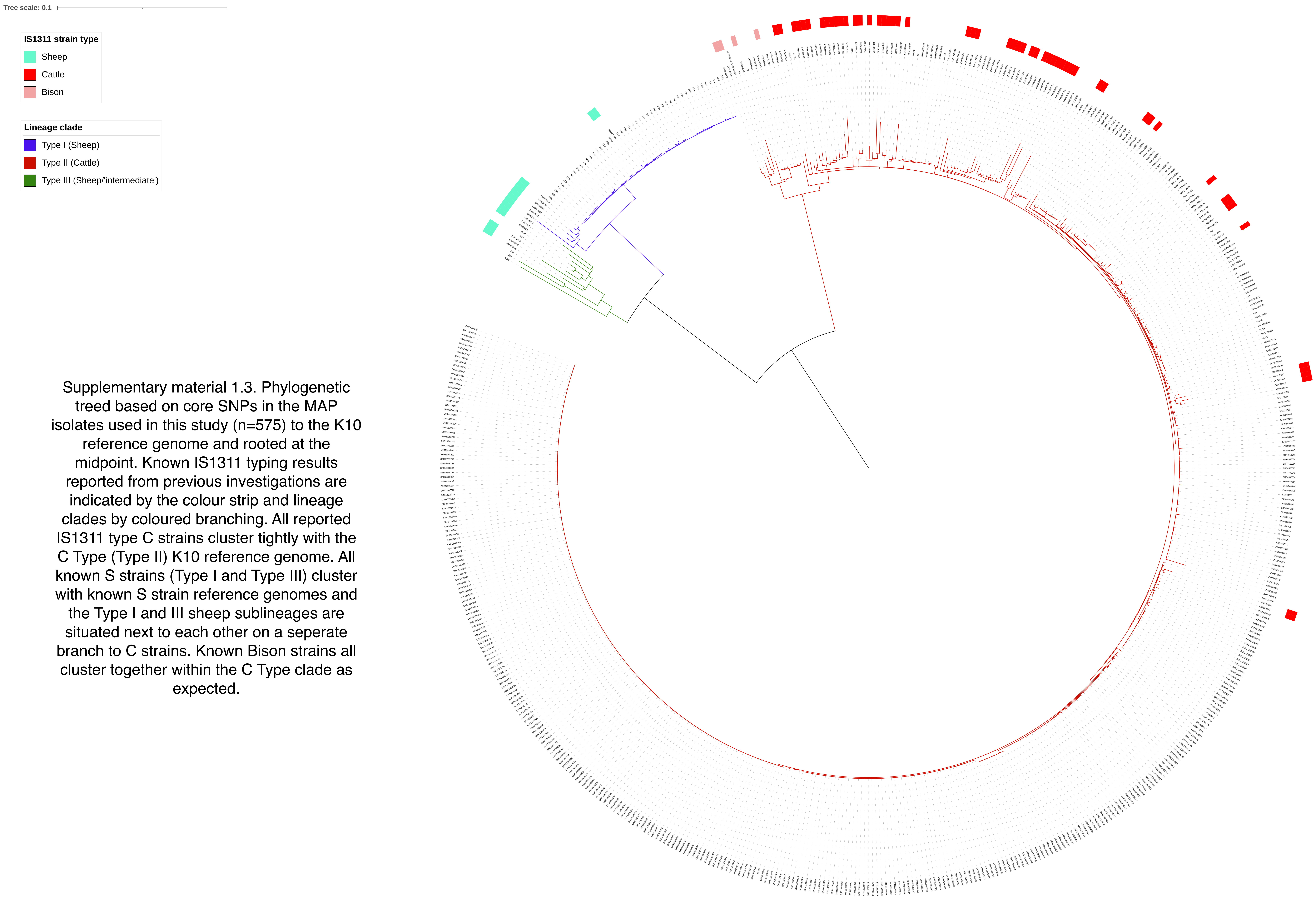

Supplementary material 1.3. Phylogenetic treed based on core SNPs in the MAP isolates used in this study (n=575) to the K10 reference genome and rooted at the midpoint. Known IS1311 typing results reported from previous investigations are indicated by the colour strip and lineage clades by coloured branching. All reported IS1311 type C strains cluster tightly with the C Type (Type II) K10 reference genome. All known S strains (Type I and Type III) cluster with known S strain reference genomes and the Type I and III sheep sublineages are situated next to each other on a seperate branch to C strains. Known Bison strains all cluster together within the C Type clade as expected.

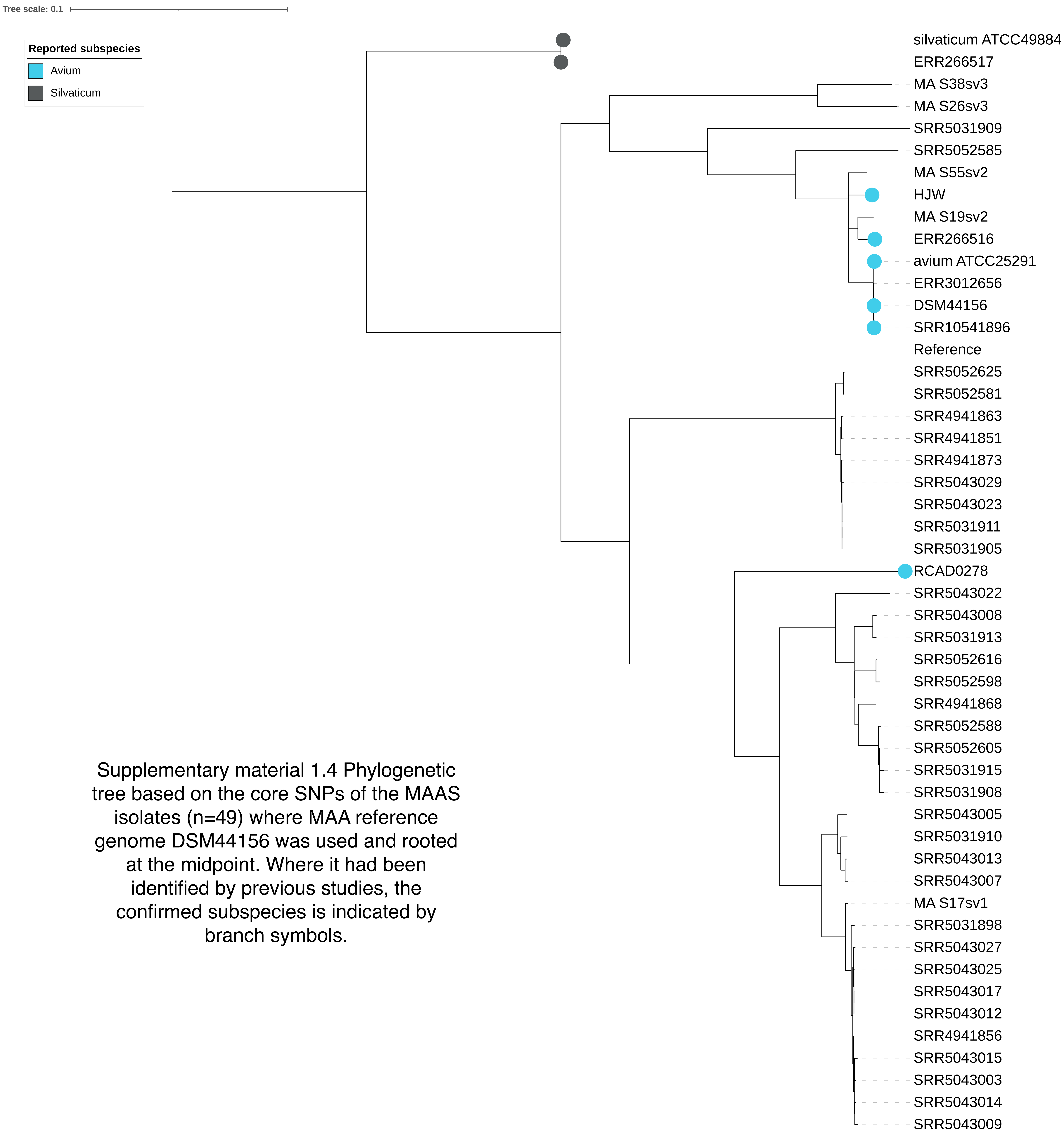

Supplementary material 1.4 Phylogenetic tree based on the core SNPs of the MAAS isolates (n=49) where MAA reference genome DSM44156 was used and rooted at the midpoint. Where it had been identified by previous studies, the confirmed subspecies is indicated by branch symbols.
